# Surgical managements of pseudoepitheliomatous keratotic and micaceous balanitis: A case report

**DOI:** 10.1016/j.ijscr.2018.12.008

**Published:** 2019-01-19

**Authors:** Joo Yeon Kim, Ji Yeon Kim, Myungchan Park, Cheol Kyu Oh, Jae-Seung Chung, Sang Hyun Park, Seong Cheol Kim

**Affiliations:** a Department of Urology, Inje University, Haeundae Paik Hospital, Busan, Republic of Korea; b Department of Pathology, Inje University, Haeundae Paik Hospital, Busan, Republic of Korea

**Keywords:** Penis, Balanitis, Treatment outcome, Penile neoplasms, Urologic surgical procedures

## Abstract

**Introduction:**

Pseudoepitheliomatous keratotic and micaceous balanitis (PKMB) is an extremely rare disease. Herein, we report a case of PKMB in a patient who underwent two surgical procedures, since the 5-FU cream was not available.

**Presentation of case:**

A 50 year-old Korean man undergoing circumcision in a local clinic presented with a tumor-like lesion on the glans penis. Peeling the mass was performed to remove the entire mass after an excisional biopsy. A pathologic finding of mass showed hyperkeratotic and papillomatous squamous epithelium without obvious cytologic atypia. Considering that the lesion recurred after 4 weeks, the patient underwent glansectomy with split-thickness skin graft (STSG). There had been no evidence of recurrence at the surgical site during the follow-up at 6 years postoperatively.

**Discussion:**

If the 5-FU cream is not available, two surgical procedures can be performed for treatment and biopsy. Peeling the mass has the advantage of confirming the characteristics of the whole lesion, but it cannot confirm tumor invasion because it is unable to obtain the subepithelial layer. Glansectomy is able to accurately identify the tumor stage because it removes the tumor and total glans penis and has excellent outcome.

**Conclusion:**

PKMB is very rare and has a characteristic appearance, which is mica-like crusts and keratotic horny mass on the glans penis. Glansectomy with STSG is a good procedure when the 5-FU cream was not available.

## Highlights

• Pseudoepitheliomatous keratotic and micaceous balanitis is rare and had the distinctive clinical findings.
• Deep biopsy is needed to diagnose the accurate tumor staging.
• Glansectomy with split-thickness skin graft can be performed to treat and diagnose.

## 1 Introduction

Pseudoepitheliomatous keratotic and micaceous balanitis (PKMB) is an extremely rare disease. The distinctive clinical findings of this disease are mica-like crusts and hard keratotic mass on the glans penis. Since its first report in 1961, less than 30 case reports have been reported worldwide [1]. Although a topical 5-fluorouracil (5-FU) treatment has been proposed as a golden standard treatment for early lesions [[2], [3], [4], [5]], surgical treatment is required if the recurrence continues despite the application of 5-FU cream or if the cream is not available [6,7]. Herein, we report a case of pseudoepitheliomatous keratotic and micaceous balanitis in a patient who underwent two surgical procedures, since the 5-FU cream was not available. We also report the patient’s surgical outcomes.

This work has been reported in accordance with the SCARE criteria [8].

## 2 Presentation of case

**Fig. 1:**
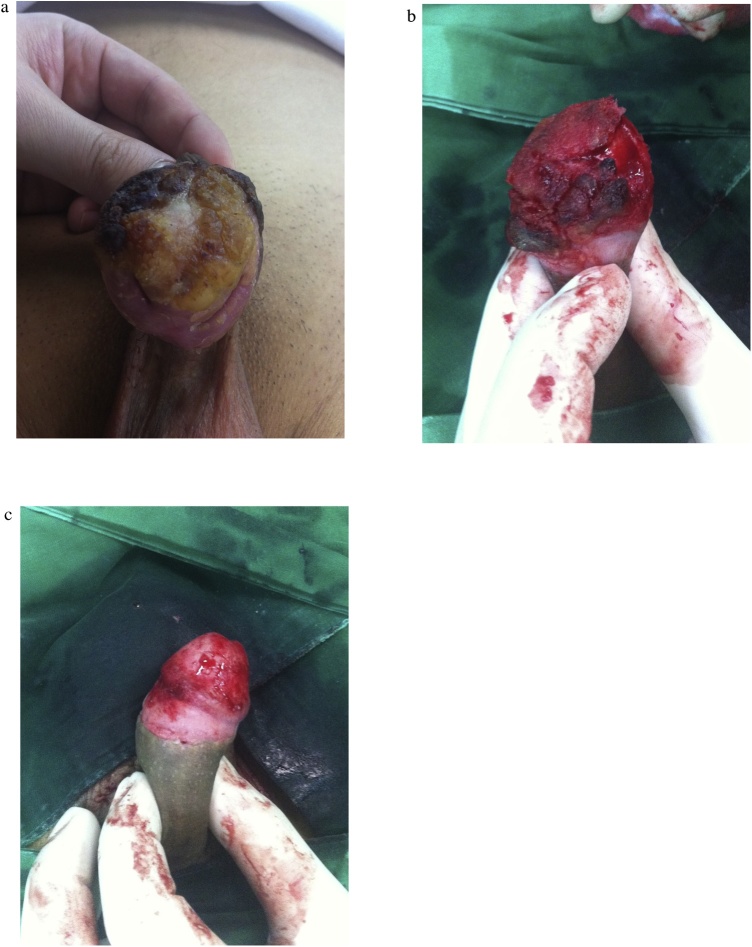
Pseudoepitheliomatous keratotic and micaceous balanitis (PKMB). (a) Mica-like crusts and hard keratotic mass found in almost the entire glans penis. (b) A hyperkeratotic plaque is easily separated from the glans penis. (c) After the complete removal of the hard mass, oozing blood is observed on the separated surface.

A 50 year-old Korean man undergoing circumcision in a local clinic because of persistent urinary tract infection presented with a tumor-like lesion on the glans. He had no pain or signs of irritation. He had no history of sexually transmitted disease (STD) and prohibited drug use, but was 15 pack-year smoker. Physical examination (Fig. 1a) showed hard, bark-like lesions located in almost the entire glans penis, but not in the urethral meatus. Lesions were localized on the glans penis, whereas the penile shaft and scrotum were normal. Inguinal lymph node was not palpable. In the blood test, serologic markers of STD were all negative. He was diagnosed as having a papillary squamous proliferative lesion, consistent with condyloma accuminatum, as confirmed by an excisional biopsy from the center of the lesion.

### 2.1 Resurfacing (peeling the mass)

**Fig. 2:**
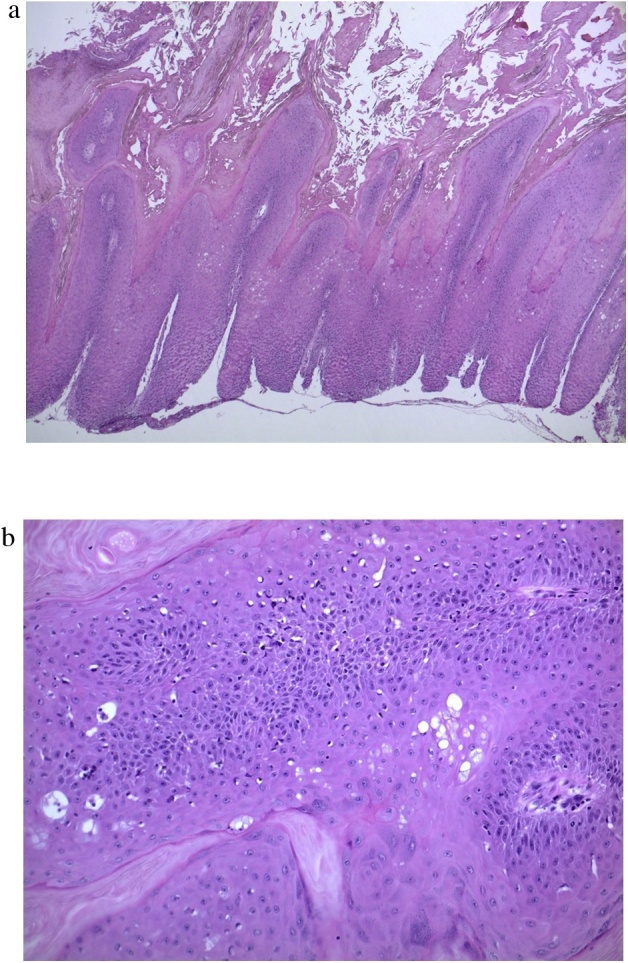
(a) Hyperkeratotic and papillomatous squamous epithelium with broad pushing margin. Subepithelial stromal tissue not obtained in this specimen (×12.5). (b) On high power view (×100), it is characterized by papillomatosis with fibrovascular cores. Keratinocytes show clear cytoplasm and winkled nuclei, also called as “koilocytosis,” which is consistent with HPV-related cytopathic changes, but not obvious cytologic atypia.

After administration of spinal anesthesia, the hard mass was dissected starting from the urethral meatus, which was easily separated from the glans penis (Fig. 1b-c). It was also separated easily from inner prepuce skin up to the circumcised scar, and oozing blood was observed on the separated surface. Pathological findings (Fig. 2) showed hyperkeratotic and papillomatous squamous epithelium without obvious cytologic atypia. Human papilloma virus (HPV) DNA and p16 was not identified through polymerase chain reaction (PCR) and immunohistochemical staining, respectively.

Epithelialization of the peeled surface took 3 weeks, and the patient complained of severe pain during penile erection and contact. Recurrence was observed in the glans after 4 weeks, and the recurred mass was observed around the glans penis and circumcised scars until 8 weeks.

### 2.2 Glansectomy with split-thickness skin graft (STSG) (Fig. 3)

Glansectomy with STSG was performed using the method reported by Parnham et al. [9]. During the operation, a frozen section from the urethral meatus was confirmed to be free from the remnant lesion. Pathological findings (Fig. 4) showed malignant cells, which showed enlarged nuclei with an irregular configuration. The tumor-stromal interface was irregular and destructive, indicating that the tumor invades to the subepithelial connective tissue. HPV type 18 was identified, but p16 was not detected. Therefore, the patient was diagnosed as having warty carcinoma out of HPV-related squamous cell carcinoma.

**Fig. 3:**
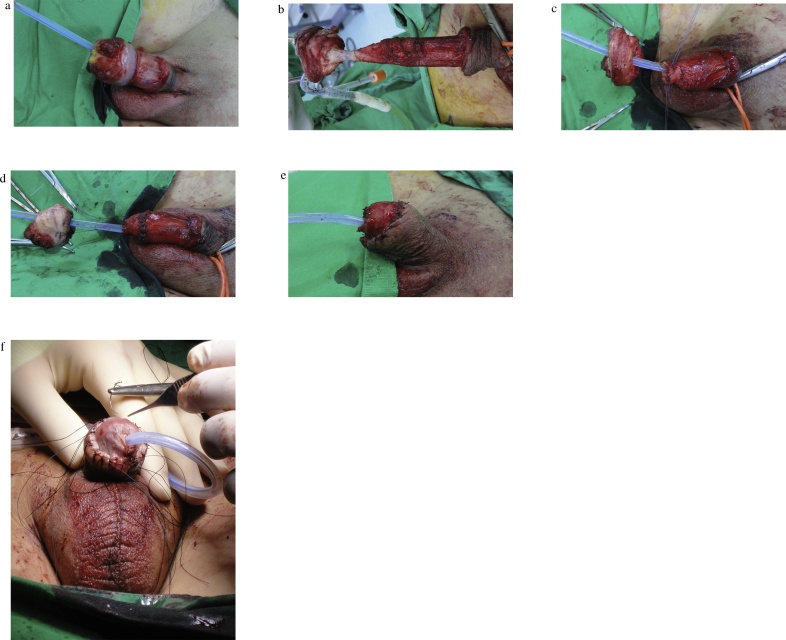
Glansectomy with split-thickness skin graft. (a) Dissection over Buck’s fascia after circumcised incision. (b) Urethra is divided to allow complete excision of the glans penis. (c) After obtaining an intraoperative frozen section at the urethral margin, the urethral margin is sutured to the corpora by using a 5-0 polyglactin suture at 12, 3, 6 and 9 o’clock positions. (d) Neurovascular bundle is ligated by using a 4-0 polyglactin suture. (e) Neoglans is created by fixing the penile shaft skin to the corpora by using a 4-0 polyglactin suture. (f) Split-thickness skin graft

**Fig. 4:**
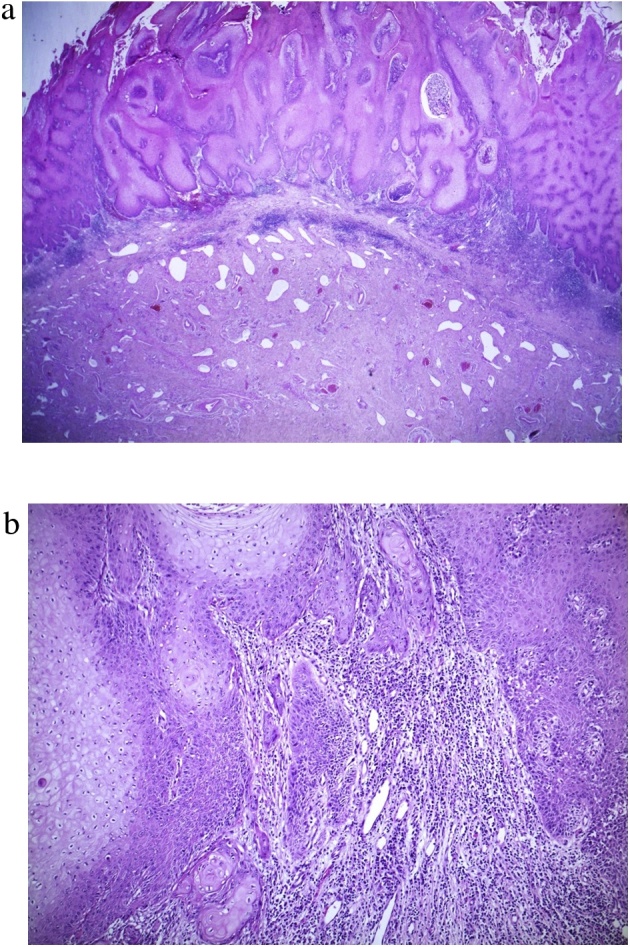
(a) There are condylomatous and rounded arborescent papillae with prominent fibrovascular cores. The superficial portion of the squamous epithelium shows well-differentiated and koilocytotic atypia (×12.5). (b) At the invasive margin in the deeper portion, the tumor-stromal interface is irregular and destructive, which is not seen in a giant condyloma. Malignant cells show enlarged nuclei with an irregular configuration, and occasionally dyskeratotic cells are noted (×100).

**Fig. 5:**
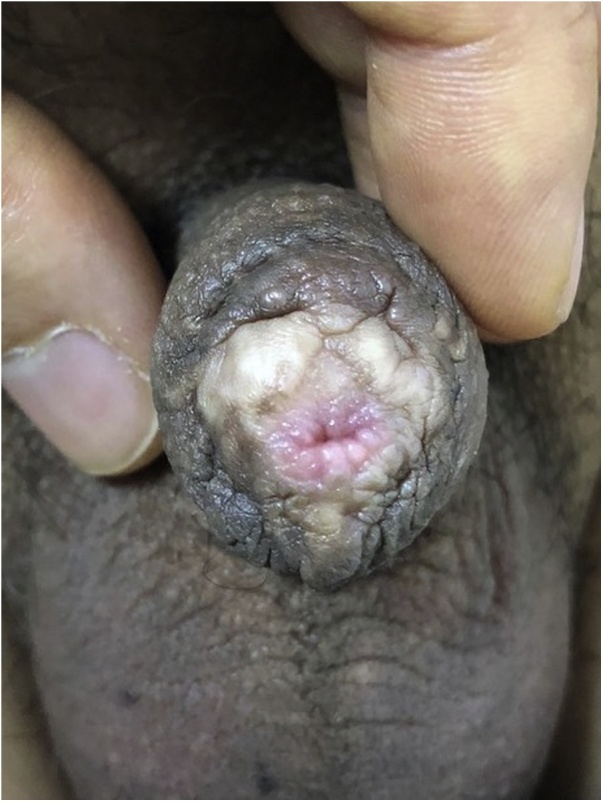
Final follow-up findings at 6 years after surgery.

There had been no evidence of recurrence at the surgical site with a follow-up duration of 6 years (Fig. 5). He complained of penile length shortening due to glansectomy, but without erectile dysfunction and voiding problems.

## 3 Discussion

PKMB is a rare disease that typically occurs on the glans penis in older men [7]. It has a very characteristic appearance, which is mica-like crusts and keratotic horny mass on the glans penis, and it is necessary to distinguish it from hypertrophic lichen sclerosis, Buschke-Lowenstein tumor, verrucous carcinoma, and squamous cell carcinoma. Characteristically, the term “keratotic and micaceous” comes from mica-like scales, which are the bark-like appearance of the lesions. The accurate etiology and pathophysiology are unknown to date.

Considering the rarity of this disease, there is a lack of research on the appropriate treatment methods. However, studies to date have suggested that the treatment methods should depend on the stage of the disease [4,7,10]. Krunic et al. [5] reported that PKMB transforms into four stages: a) initial plaque stage, b) late tumor stage, c) verrucous carcinoma, and d) squamous cell carcinoma and invasion. Based on these stages, a topical therapy using a 5-FU cream is recommended for the initial plaque stage or late tumor stage, and surgical resection for verrucous carcinoma or squamous cell carcinoma.

Most previous studies recommend the topical therapy, but in countries where such agent is not available, wide excision may be considered such as partial penectomy [11]. Among the recent reports of penile cancer, glansectomy with STSG for tumors confined in the glans penis has been reported to have excellent results in terms of cosmetic or functional outcomes [9]. Another surgical option is resurfacing using the property of easy to be peeled off if the patient wants to preserve his glans penis. However, this surgical procedure has the following disadvantages: (1) difficulty in obtaining subepithelial tissues, which are needed to confirm invasiveness; (2) causes severe pain during the epithelialization period.

In this case, three methods were used to obtain tissues to confirm the pathological findings. First, excisional deep biopsy, which is performed to determine the direction of treatment; however, this cannot identify exactly the stage of the entire tumors, because only a part of the tumor is identified. Second, resurfacing has the advantage of confirming the characteristics of the whole lesion because it removes the entire tumors, but it cannot confirm tumor invasion because it is unable to obtain the subepithelial layer. Lastly, glansectomy is able to accurately identify the stage, including tumor invasion, because it removes the tumor and total glans penis. However, this procedure has several disadvantages. It may be more invasive and requires additional skin graft, and patients may be dissatisfied with the appearance of the penis after the surgery.

PKMB is often resistant to topical treatments, such as 5-FU cream, radiotherapy and cryotherapy. In these cases, progression to verrucous carcinoma or squamous cell carcinoma has been reported in a considerable proportion of cases, and they are now considered tumors with a malignant potential [7]. Considering that excisional biopsy is unable to confirm malignant cells in the entire lesion, patients with poor response to topical therapy require more aggressive treatments, such as glansectomy.

## 4 Conclusion

PKMB is very rare and has a characteristic appearance, which is mica-like crusts and keratotic horny mass on the glans penis. Deep biopsies are needed to obtain subepithelial tissues for the accurate diagnosis of PKMB. Glansectomy with STSG is a good procedure when the 5-FU cream was not available.

## Conflict of interest

The authors report no conflict of interests.

## Funding source

There was no funding.

## Ethical approval

This case report was approved by institutional review board in our institution (IRB No. 2017-12-008).

## Consent

Written informed consent was obtained from the patient for publication of this case report and accompanying images. A copy of the written consent is available for review by the Editor-in-Chief of this journal on request.

## Author contribution

Seong Cheol Kim: Operated the patient, Wrote the manuscript, Drafted the manuscript.

Joo Yeon Kim, Ji Yeon Kim: Diagnosed the tumors pathologically.

Cheol Kyu Oh, Jae-Seung Chung: Reviewed the manuscript.

Myungchan Park, Sang Hyun Park: Collected the data.

## Registration of research studies

None.

## Guarantor

Seong Cheol Kim.

## Provenance and peer review

Not commissioned, externally peer-reviewed.
